# Schistosoma species detection by environmental DNA assays in African freshwaters

**DOI:** 10.1371/journal.pntd.0008129

**Published:** 2020-03-23

**Authors:** Hind Alzaylaee, Rupert A. Collins, Gabriel Rinaldi, Asilatu Shechonge, Benjamin Ngatunga, Eric R. Morgan, Martin J. Genner

**Affiliations:** 1 School of Biological Sciences, University of Bristol, Life Sciences Building, Bristol, United Kingdom; 2 Faculty of Sciences, Biology Department, Prince Nourah Bin Abdulrahman University, Riyadh, Saudi Arabia; 3 Wellcome Sanger Institute, Wellcome Genome Campus, Hinxton, United Kingdom; 4 Tanzania Fisheries Research Institute (TAFIRI), Dar es Salaam, Tanzania; 5 School of Biological Sciences, Queen’s University Belfast, Belfast, United Kingdom; University of Pennsylvania, UNITED STATES

## Abstract

**Background:**

Schistosomiasis is a neglected tropical parasitic disease associated with severe pathology, mortality and economic loss worldwide. Programs for disease control may benefit from specific and sensitive diagnostic methods to detect *Schistosoma* trematodes in aquatic environments. Here we report the development of novel environmental DNA (eDNA) qPCR assays for the presence of the human-infecting species *Schistosoma mansoni*, *S*. *haematobium* and *S*. *japonicum*.

**Methodology/Principal findings:**

We first tested the specificity of the assays across the three species using genomic DNA preparations which showed successful amplification of target sequences with no cross amplification between the three focal species. In addition, we evaluated the specificity of the assays using synthetic DNA of multiple *Schistosoma* species, and demonstrated a high overall specificity; however, *S*. *japonicum* and *S*. *haematobium* assays showed cross-species amplification with very closely-related species. We next tested the effectiveness of the *S*. *mansoni* assay using eDNA samples from aquaria containing infected host gastropods, with the target species revealed as present in all infected aquaria. Finally, we evaluated the effectiveness of the *S*. *mansoni* and *S*. *haematobium* assays using eDNA samples from eight discrete natural freshwater sites in Tanzania, and demonstrated strong correspondence between infection status established using eDNA and conventional assays of parasite prevalence in host snails.

**Conclusions/Significance:**

Collectively, our results suggest that eDNA monitoring is able to detect schistosomes in freshwater bodies, but refinement of the field sampling, storage and assay methods are likely to optimise its performance. We anticipate that environmental DNA-based approaches will help to inform epidemiological studies and contribute to efforts to control and eliminate schistosomiasis in endemic areas.

## Author summary

Schistosomiasis, otherwise known as bilharzia or snail fever, is a prevalent human disease found across tropical regions of the world and is a major cause of disability. The disease is acquired from exposure to the schistosome infectious larvae released by infected host snails in freshwaters. Programs to restrict the transmission of schistosomiasis would benefit from rapid and reliable diagnostic methods to detect schistosomes. Here we report a study that has developed new diagnostic tools to identify the DNA from three human-infecting *Schistosoma* species within water samples. This “environmental DNA” (eDNA) approach requires the filtering and laboratory analyses of water samples, and avoids the requirements to locate, identify and individually test the infectious status of host snails. Our results showed that eDNA methods detect the presence of the parasite in freshwater bodies. However, there is need for further refinement for sampling and laboratory techniques to improve the performance of the assays. We anticipate that eDNA approaches will provide information on the distribution and abundance the water-borne parasites, and potentially contribute to the control and elimination of schistosomiasis.

## Introduction

Schistosomiasis, a neglected tropical disease caused by trematodes of the genus *Schistosoma*, is the second most prevalent parasitic disease in humans, after malaria [1, 2]. Studies have indicated that upwards of 207 million people may be infected globally, with a further 779 million individuals at risk of infection [2]. Around 93% of infected people live in sub-Saharan Africa [2, 3], where widespread poverty and the absence of clean water and sanitary facilities contribute to the incidence and propagation of the disease [4, 5]. Schistosomiasis is associated with health problems including malnutrition, anaemia, and impaired childhood development. It is also a major impediment to education, health nutrition and socio-economic development in the infected populations [1, 6, 7, 8].

There are three main *Schistosoma* species that infect humans. *S*. *haematobium* causes urogenital schistosomiasis and is present across Africa and the Middle East. *S*. *mansoni*, the agent of intestinal schistosomiasis, is prevalent across Africa, the Middle East and South America. *S*. *japonicum* causes hepatic schistosomiasis and is mainly found in the Philippines, Indonesia and China. As trematodes, *Schistosoma* species have a complex life cycle that requires an intermediate freshwater snail host and a final vertebrate host [1, 8]. Snails that are responsible for schistosomiasis in humans belong to one of three genera: *Biomphalaria* (for *S*. *mansoni)*, *Bulinus* (for *S*. *haematobium*) and *Oncomelania* (for *S*. *japonicum*) [8]. Transmission to humans occurs when the free-swimming larval form (cercaria) is shed from infected freshwater snails and penetrates the skin of the definitive host [8, 9]. Once mature, the male and female schistosomes mate, reproduce, and produce eggs that are excreted via urine or faeces, depending on the species. Eggs excreted into freshwater environments hatch and release mobile miracidia which infect snails to continue the cycle [8].

Awareness of safe water sources by human populations in endemic regions is critical for prevention of schistosomiasis [10, 11]. However, assessment and therefore mitigation of the risk posed by bodies of freshwater is not straightforward. Despite large integrated control programs during the last few decades, disease transmission continues due to the absence of rapid and reliable diagnostic tools to detect the schistosomes in endemic areas [12]. Historically the presence of schistosomes has been established by snail-based surveys coupled with microscopic and/or molecular detection of parasitic larvae within the snails [13–18]. These surveys are labour intensive and demand specific training and expertise. Moreover, the sensitivity of this method is generally low given that snail infection rates can be as low as 1–2% of the population, even where there is a high prevalence of infected humans [19, 20].

Over recent years conservation biologists and ecologists have widely adopted the use of environmental DNA (eDNA) methods to establish the presence of species that are difficult to study using other approaches [21–27]. These methods can be particularly useful for studying parasites because they can be challenging to locate and reliably identify. From a parasitological perspective, the term eDNA can be defined as “DNA extracted from an environmental or organismal matrix” [28]. Specifically, this definition includes target DNA from whole microscopic organisms present in the sample, and is not restricted to DNA in solution or cellular debris [28]. Successful detections of trematode parasites using aquatic eDNA include *Ribeiroia ondatrae* in North America [29], *Opisthorchis viverrini* in southeast Asia [30], and *Fasciola hepatica* and *Calicophoron daubneyi* in Europe [31]. Previous studies have also shown the feasibility of recovering *Schistosoma mansoni* DNA from both field and laboratory environments [32, 33]. They have also demonstrated that *Schistosoma* DNA present in laboratory water decayed below levels of detection eight days after the removal of source snails [33]. Collectively, this evidence is highly supportive of the potential of eDNA methods for improved surveillance of all harmful schistosome species [34], but also indicates that improvements are needed to adapt these methods to large-scale surveillance, and to identify species other than *S*. *mansoni*.

In this study, we built on previous studies by developing novel eDNA methods to detect human *Schistosoma* in freshwater bodies. Specifically, we report the development of primers and probes for the mitochondrial 16S rRNA region for *S*. *mansoni*, *S*. *haematobium* and *S*. *japonicum*, and undertake thorough tests of assay specificity. We then validated sensitivity of the *S*. *mansoni* assay by testing eDNA from water collected from aquaria holding infected host snails. Finally, we evaluated the applicability and sensitivity of the new assays to detect *S*. *mansoni* and *S*. *haematobium* eDNA in Tanzanian freshwaters where both species are co-endemic.

## Materials and methods

### Primer and probe design

Species-specific sets of primers and probes were developed to target short (< 150 basepair) fragments of the mitochondrial DNA 16S rRNA gene in *S*. *japonicum*, *S*. *mansoni* and *S*. *haematobium*. The following GenBank sequences were used as templates: *S*. *mansoni* HE601612, *S*. *haematobium* DQ157222 and *S*. *japonicum* JQ781206. Sequences were aligned to available mitochondrial 16S rRNA sequences of eight other *Schistosoma* species using ClustalW in Bioedit v.7.2.6 [35], namely *Schistosoma curassoni* AP017708, *Schistosoma mekongi* AF217449, *Schistosoma margrebowiei* AP017709, *Schistosoma spindale* DQ157223, *Schistosoma bovis* QMKO01004774, *Schistosoma rodhaini* LL973454, *Schistosoma indicum* EF534284 and *Schistosoma incognitum* EF534285. We used PrimerBlast [36] and PrimerQuest (Integrated DNA Technologies, Coralville IA; https://eu.idtdna.com/Primerquest/Home/Index) to design primers and probes. In both tools we selected default settings, except PCR product size was restricted to between 70 to 170 bp. To avoid cross-species amplification, primers were selected that had at least two mismatches with non-target species. Probes were designed with a dual labelled 5(6)-carboxy-fluorescein (FAM) fluorescent tag at the 5’ end and with a Black Hole Quencher 1 at the 3’ end (Integrated DNA Technologies, Coralville, IA). Final expected lengths of PCR products for *S*. *mansoni*, *S*. *haematobium* and *S*. *japonicum* were 104, 143 and 87 bp, respectively (Table 1).

**Table 1:** Species-specific primers and probes designed for PCR amplification of the 16S rRNA mitochondrial gene from three human-infecting *Schistosoma* species.

Target species	Primers / Probe	Nature	Sequences (5’-3’)	Product length (bp)
*Schistosoma mansoni*	SM-16SrRNA-F	Forward	CTGCTCAGTGAAGAAGTTTGTTT	104 bp
	SM-16SrRNA-P	Probe	AGCCGCGATTATTTATCGTGCTAAGGT	
	SM-16SrRNA-R	Reverse	CCTCATTGAACCATTCACAAGTC	
*Schistosoma haematobium*	SH-16SrRNA-F	Forward	AATGAACATGAATGGCCGCA	143 bp
	SH-16SrRNA-P	Probe	TGGAGACTTGTGAATGGTCGAACG	
	SH-16SrRNA-R	Reverse	ATGGGTTCCTCACCACTTAAACT	
*Schistosoma japonicum*	SJ-16SrRNA-F	Forward	TATGGCCTGCCCAATGTTGT	87 bp
	SJ-16SrRNA-P	Probe	TGGTCGCAGTTTTACTGTGCTAAGGT	
	SJ-16SrRNA-R	Reverse	ACAAGCCACTAATTAAGAAGCGA	

### Specificity of *S*. *mansoni*, *S*. *haematobium* and *S*. *japonicum* assays

**Fig 1:**
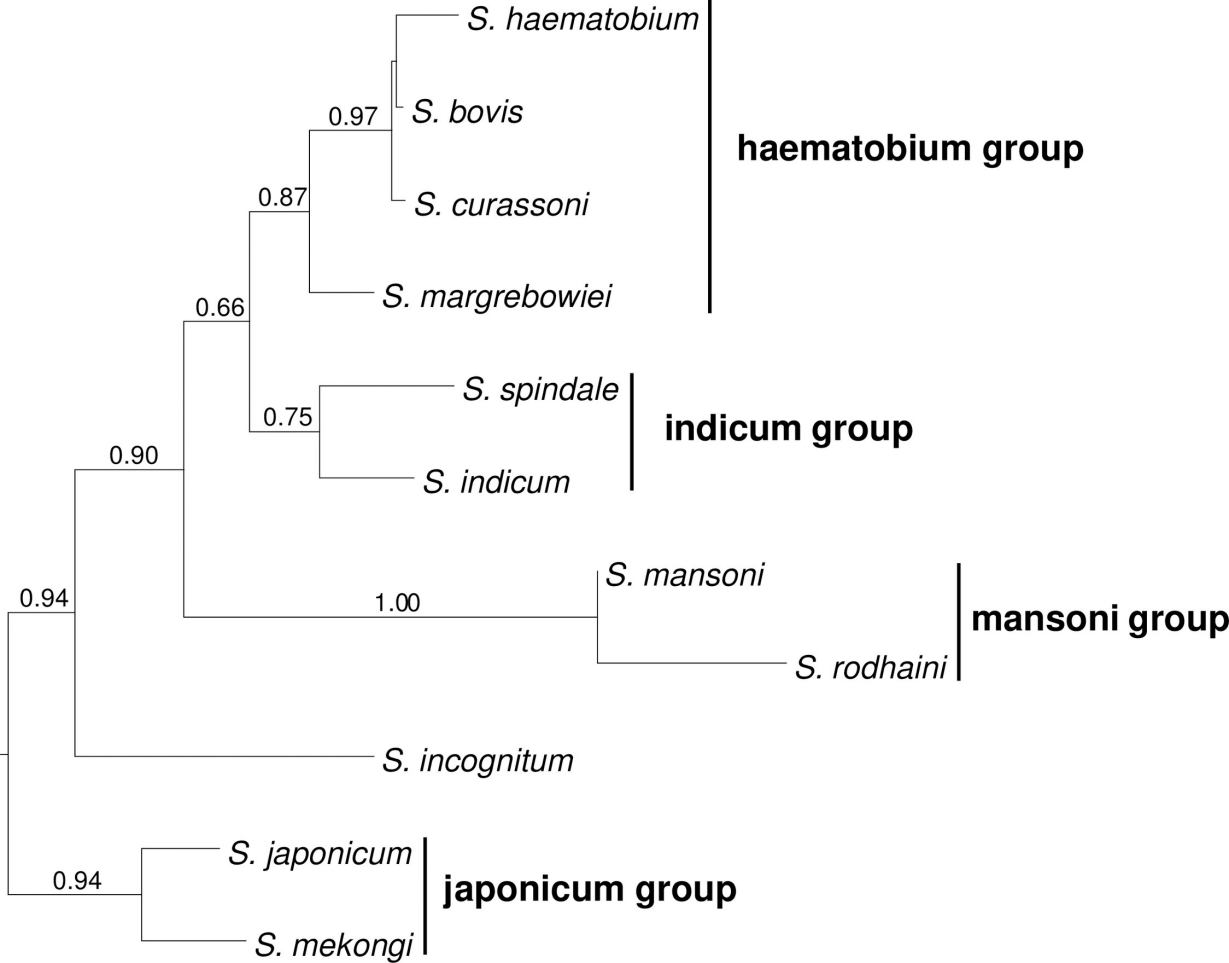
Maximum likelihood phylogenetic reconstruction of the mtDNA 16S sequences used for the establishing the specificity of the assays designed for *S*. *haematobium*, *S*. *mansoni* and *S*. *japonicum*. Numbers above branches represent the branch support (proportion of 100 bootstrap replicates.

Specificity of the primer/probe sets was established using quantitative PCR (qPCR) tests on both genomic and synthetic DNA of known origin. Genomic DNA from *S*. *mansoni*, *S*. *haematobium* and *S*. *japonicum* was sourced from BEI Resources Repository of the National Institute of Allergy and Infectious Diseases (NIAID, Manassas, VA, USA), and diluted to 0.01ng/μl for qPCR tests. The synthetic DNA was generated from GenBank published sequences for *S*. *mansoni*, *S*. *haematobium*, *S*. *japonicum*, *S*. *curassoni*, *S*. *margrebowiei*, *S*. *spindale*, *S*. *bovis*, *S*. *rodhaini*, *S*. *indicum*, *S*. *incognitum* and *S*. *mekongi* (Table 2). Synthesis was carried out by Eurofins Genomics (Ebersberg, Germany) within a pEX-A128 *E*. *coli* vector, and Invitrogen by Thermo-Fisher Scientific (Waltham, MA, USA) within a pMA-T *E*. *coli* vector or pMA-RQ (AmpR) *E*. *coli* vector. Accession numbers, fragment lengths and total synthesised DNA concentration are shown in S1 Table. To visualise the evolutionary relationships of these mitochondrial sequences, they were aligned using Clustalw in DAMBE7 [37] and a maximum likelihood phylogeny was generated using the HKY+Γ model in Mega-X [38] with branch support estimated from 100 bootstrap replicates (Fig 1).

**Table 2:** Tests of *S*. *mansoni*, *S*. *haematobium and S*. *japonicum* qPCR assays on the genomic (gen) and synthetic (syn) DNA of *Schistosoma* species.

Target DNA			Primers	
		*S*. *mansoni* Ct mean (range)	*S*. *haematobium* Ct mean (range)	*S*. *japonicum* Ct mean (range)
*S*. *mansoni* (gen)	0.01 ng	27.88 (27.79–28.03)	-	-
*S*. *haematobium* (gen)	0.01 ng	-	28.71 (28.54–28.87)	-
*S*. *japonicum* (gen)	0.01 ng		-	26.83 (26.49–27.01)
*S*. *mansoni* (syn)	1000 copies/μl	29.38 (28.94–30.17)	-	-
*S*. *rodhaini* (syn)	1000 copies/μl	-	-	-
*S*. *japonicum* (syn)	1000 copies/μl	-	-	28.47 (28.06–28.84)
*S*. *mekongi* (syn)	1000 copies/μl	-	-	34.94 (34.61–35.35)
*S*. *indicum* (syn)	1000 copies/μl	-	-	-
*S*. *spindale* (syn)	1000 copies/μl	-	-	-
*S*. *incongnitum* (syn)	1000 copies/μl	-	-	-
*S*. *haematobium* (syn)	1000 copies/μl	-	27.93 (27.72–28.20)	-
*S*. *bovis* (syn)	1000 copies/μl	-	35.67 (35.61–35.71)	-
*S*. *curassoni* (syn)	1000 copies/μl	-	30.59 (30.43–30.72)	-
*S*. *margrebowiei* (syn)	1000 copies/μl	-	-	-

The template DNA concentration (copies of synthetic template DNA) for each qPCR reaction was calculated using a copy number and dilution calculator (Thermo-Fisher, Waltham, MA, USA; https://bit.ly/2JPZchd). Each total reaction volume of 5 μl per reaction included: 1 μl DNA extract, 2.5 μl Master Mix (PrimeTime Gene Expression Master Mix; IDT), 1.25 μl molecular grade water and 0.25 μl of primer/probe mix. The primer/probe mix included 4 μl of each primer (100 μM, IDT) and 2 μl of probe (100 μM, IDT) and 40 μl of molecular grade water. Thermocycling conditions were 95°C for 3 min, followed by 45 cycles of 95°C for 0.05 seconds and 60°C for 30 seconds. In addition to test samples, negative no-template control samples were included for quality assurance. The reactions were run on an Eco48 real time qPCR system (PCRmax, Staffordshire, UK) in 48-well plates with ROX normalisation. DNA detection was expressed by cycle threshold (Ct) values.

### Sensitivity of *S*. *mansoni* and *S*. *haematobium* assays

We tested the sensitivity of *S*. *mansoni* and *S*. *haematobium* assays, but not the *S*. *japonicum* assay. To make a quantified stock solution for qPCR, the target 16S rRNA region was first amplified from genomic DNA extracts of *S*. *mansoni* and *S*. *haematobium* sourced from BEI Resources Repository of the NIAID using the species-specific primers described above, and a conventional end-point PCR. Each PCR comprised 5μl polymerase buffer, 3μl MgCl_2_ of 25 mM, 0.2μl GoTaq DNA polymerase (5U/ml) (Promega, Madison, WI, USA), 0.5μl dNTPs (10mM each), 12.3 μl molecular grade water, 1μl of each 10 μM primer (IDT) and 2μl template DNA (total volume of 25μl per reaction). The PCR conditions were: 95°C for 2 min of initial denaturation, followed by 40 cycles of 95°C for 30 sec, 55°C for 30 sec, and 72°C for 30 sec, and a final extension at 72°C for 5 min. A negative no-template control was included. The presence of a single PCR product of expected size was confirmed on an 1.5% agarose gel stained with gel red nucleic acid gel stain, 10,000X (Biotium, Fremont, CA) and a PCRSizer 100bp DNA Ladder (Norgen Biotek, Thorold, Canada).

To make the serial dilutions to measure assay sensitivity, each PCR product was then purified using the QIAquick PCR purification kit (Qiagen) following the manufacturer’s protocol. Then, the concentrations of DNA were measured using a Qubit fluorometer (Invitrogen, Waltham, MA). The number of target copies was calculated using the Thermo-Fisher DNA copy number and dilution calculator (above). Then, the stock solution was stored at -20°C for qPCR tests. Before each qPCR test, a dilution series of known concentrations ranging from 1,000,000 copies/μl decreasing ten-fold down to 1 copy/μl was prepared for each assay (Table 3). These serial dilutions were used within two days to limit potential effects of DNA degradation.

**Table 3:** Ten-fold serial dilutions used in the *S*. *mansoni* and *S*. *haematobium* assays. In total these reflect the results of 17 *S*. *mansoni* assays, and 12 *S*. *haematobium* assays.

Assay	Concentration (copies)	Amplification success (number of individual PCRs)	Amplification success (at least one qPCR of three replicates)	Mean CT (Range) cycle	Efficiency range (%)	*r*^2^ range
*S*. *mansoni*	1,000,000	51/51	17/17	21.01 (20.08–22.32)	91.6–110.9	0.97–0.99
	100,000	51/51	17/17	24.44 (23.33–25.55)		
	10,000	51/51	17/17	27.40 (26.31–28.44)		
	1000	51/51	17/17	31.10 (30.02–32.84)		
	100	51/51	17/17	34.27 (32.44–36.67)		
	10	32/51	16/17	37.09 (35.38–42.02)		
	1	2/51	2/17	37.30 (37.06–37.55)		
*S*. *haematobium*	1,000,000	36/36	12/12	18.35 (16.94–21.86)	94.9–105.5	0.98–0.99
	100,000	36/36	12/12	21.68 (20.22–25.21)		
	10,000	36/36	12/12	25.09 (23.61–28.46)		
	1000	36/36	12/12	28.37 (24.93–32.41)		
	100	34/36	12/12	31.54 (30.06–37.72)		
	10	29/36	11/12	34.24 (32.87–37.73)		
	1	18/36	9/12	36.73 (34.98–38.17)		

Three qPCR technical replicates were run for each ten-fold standard dilution, including negative no-template controls, using the qPCR protocol described above. The limit of detection within a single qPCR reaction (LOD_I_) was defined as the lowest concentration where there is a 95% chance of amplification success in any one individual PCR reaction. We also calculated the lowest concentration with a 95% chance of amplification success in any one of three technical replicate qPCR reactions of the same sample (LOD_III_), which we considered a useful metric given that qPCR reactions of eDNA are typically conducted in triplicate. These concentration limits were derived from all standards run and calculated by fitting logistic models [39] using CurveExpert Basic 2.1.0 (Hyams Development). The limit of quantification (LOQ) was defined as the lowest concentration at which 90% of all standards run were able to be amplified, following protocols described in [40].

### Environmental DNA from aquarium water samples

To validate the accuracy of the *S*. *mansoni* qPCR assay, water samples were collected from four aquaria housing *Biomphalaria glabrata* host snails infected with *S*. *mansoni*, at the Wellcome Sanger Institute (WSI), Cambridgeshire, UK. The complete life cycle of *Schistosoma mansoni* (NMRI strain) is maintained at the WSI by breeding and infecting *B*. *glabrata* snails and mice. Mouse infections are performed under the Home Office Project Licence No. P77E8A062 held by GR. We did not specifically quantify the number or biomass of infected snails in aquaria, but the total number of snails in each aquarium was recorded. Additionally, water samples were collected from two aquaria holding non-infected *B*. *glabrata*, and a sample of sterile water was collected at the site for use as a negative control. Sampled water was filtered through a Sterivex filter with a pore size 0.22 μm and a polyethersulfone membrane (EMD Millipore corporation, UK), using a peristaltic pump unit. Volumes of water filtered in a single replicate varied between 60–500 ml (Table 4). After filtration, absolute ethanol was pushed through the filter with a sterile 50 ml syringe to preserve the samples, and each filter was kept individually in a labelled 118 ml capacity Whirl-Pak bag (Sigma-Aldrich, UK). Filters were placed in a polystyrene box or a cooler with ice during transportation to the laboratory, and kept at -20°C until being processed. Extraction of eDNA from the individual filter was performed using the DNeasy Power Water Kit (Qiagen, UK) following the manufacturer’s protocol, but eluting into 50 μl of EB buffer. The *S*. *mansoni* DNA was quantified using qPCR procedures as described above for the specificity assays. Three PCR replicates were carried out for each sample, alongside three replicates of a negative no-template PCR control, and three replicates of each concentration in a standard curve serial dilution of control positive PCR-derived DNA of *S*. *mansoni* (ranging from 1,000,000 copies/μl to 1 copy/μl).

**Table 4:** Detection of *S*. *mansoni* DNA from aquarium water samples.

Status	Replicate	No. snails (individuals)	Water filtered (ml)	DNA concentration ng per μl	Amplification success	Ct mean (cycle)	Mean copies per μl (range)
Non-infected	Tank 1: Rep 1	33	500	54.95	0/3	-	-
	Tank 1: Rep 2		460	58.50	0/3	-	-
	Tank 2: Rep 1	28	500	115.00	0/3	-	-
	Tank 2: Rep 2		460	61.3	0/3	-	-
Infected	Tank 3: Rep 1	19	160	84.50	3/3	26.95	9041 (8580 to 9949)
	Tank 3: Rep 2		90	63.65	3/3	27.77	9011 (8392 to 9958)
	Tank 4: Rep 1	75	200	85.90	3/3	27.19	7565 (7342 to 7737)
	Tank 4: Rep 2		200	114.10	3/3	26.34	14038 (13384 to 14518)
	Tank 5: Rep 1	13	70	88.95	3/3	25.58	24599 (22498 to 28659)
	Tank 5: Rep 2		60	102.20	3/3	25.39	28130 (26232 to 30259)
	Tank 6: Rep 1	18	73	100	3/3	25.15	33296 (33066 to 33739)
	Tank 6: Rep 2		78	114.30	3/3	24.64	48264 (45033 to 52181)
Negative control	NA	0	500	-	0/3	-	-

### Environmental DNA from the natural environment

**Fig 2:**
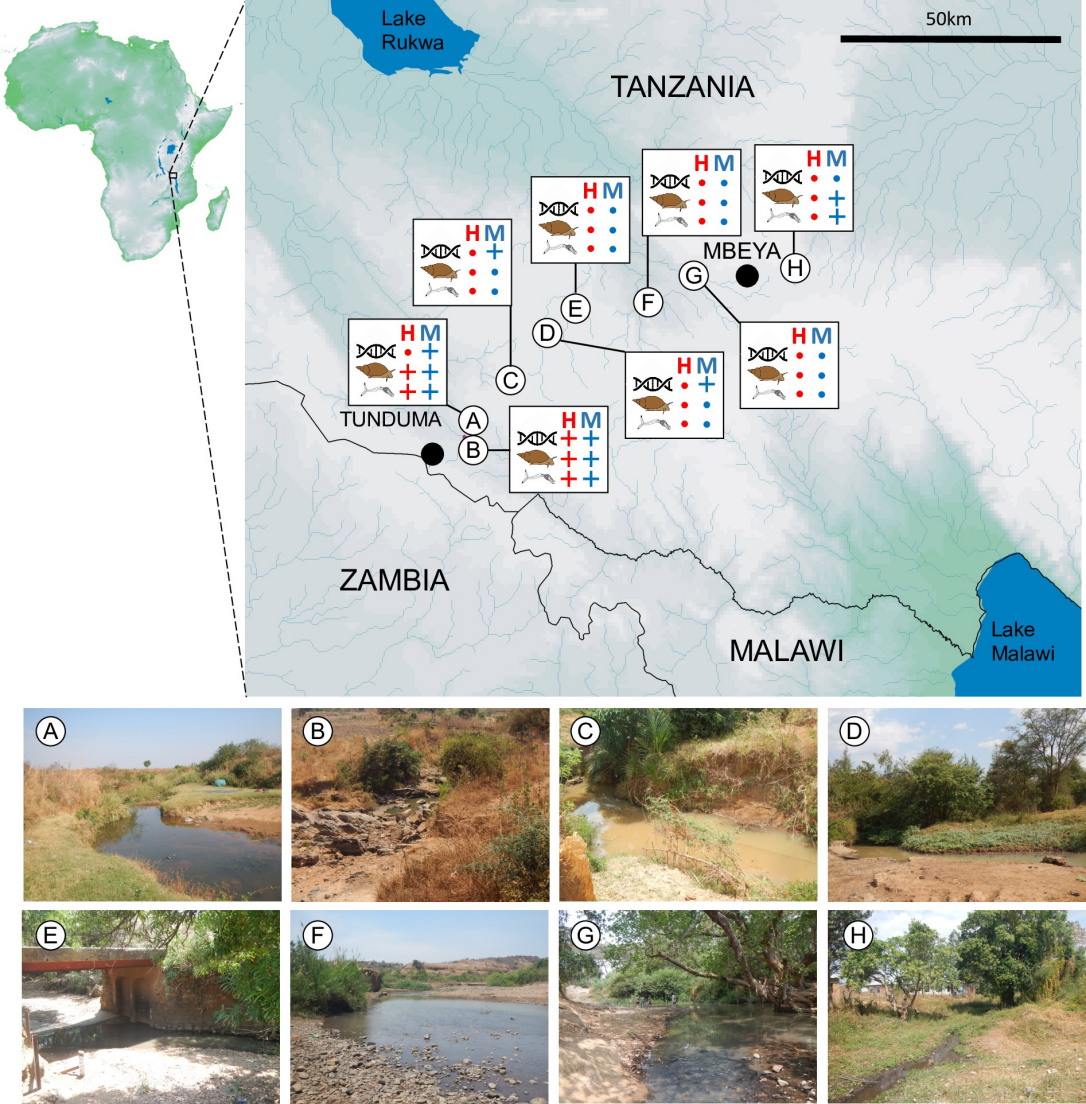
Locations of eDNA sampling sites in the Mbeya Region of Tanzania in 2018. In total eight sites were surveyed, labelled A-H. The key results from eDNA survey (helix symbol), host snail survey (snail symbol) and test for the presence of schistosomes in snail tissue (cercariae symbol) are given for *S*. *haematobium* (H) and *S*. *mansoni* (M) assays, with + indicating the site was positive, while a dot indicates no detection was made. Map drawn using the following open source software and data: DIVA-GIS7.5 (https://www.diva-gis.org); Africa river network (15 sec resolution), African drainage basin (15sec resolution) and African Digital Elevation Model data (30 sec resolution) from HydroSHEDS (https://www.hydrosheds.org); African Water Bodies shapefile from RCMRD GeoPortal (http://geoportal.rcmrd.org).

To test the *S*. *mansoni* and *S*. *haematobium* assays on samples from natural habitats, water samples were collected from the surface of freshwaters at eight locations in Tanzania during September 2018 (Table 5; Fig 2). These included locations where both *S*. *mansoni* and *S*. *haematobium* were plausibly co-endemic due to the presence of host snails and close proximity of human settlements. It also included locations where both *S*. *mansoni* and *S*. *haematobium* were plausibly absent due to the absence of host snails and the greater distance from settlements. At each location coordinates were obtained using a handheld etrex GPS (Garmin, Olathe, KS), and the pH, conductivity, total dissolved solids and temperature of the water were measured using a multimeter (Hach, Loveland, CO) (Table 5). At each location MJG conducted a 10 minute search for aquatic snails in shallow water benthic habitats including rock surfaces, soft sediments and marginal vegetation. The collected snails from each site were preserved in absolute ethanol and stored in 118 ml capacity whirlpak bags. Additional samples were also collected at sites A and B allowing more thorough testing of the frequency of infected snails at these sites (Table 5).

**Table 5:** Sampling locations and environmental characteristics of eDNA from Tanzania in 2018.

Site.	Name	Latitude (decimal degrees)	Longitude (decimal degrees)	Sampling date	Maximum water depth (m)	Total dissolved solids (ppm)	Conductivity (μS/m)	Temperature (˚C)	pH	*Bulinus globosus* (Individuals per 10 min search)	*Biomphlaria pfeifferi* (Individuals per 10min search)	*Lymnaea natalensis* (Individuals per 10min search)	Infected *Bulinus* / total tested by PCR	Infected *Biomphalaria* / total tested by PCR
A	Mpemba River	-9.24289	32.84196	16-Sep-18	1	205	300	23.3	8.15	4	133	1	29 / 48	145 / 364
B	Mpemba River	-9.26564	32.84167	16-Sep-18	0.5	288	430	27.5	8.67	55	36	1	49 / 52	14 / 16
C	Vwawa River	-9.12298	32.91646	16-Sep-18	0.5	57	86	21.8	7.82	0	0	0	abs	abs
D	Mlowo River	-9.01295	33.01048	16-Sep-18	0.5	106	114	22.4	7.56	0	0	1	abs	abs
E	Myovizi River	-8.97372	33.0663	17-Sep-18	0.5	90	135	18.6	8.40	0	0	0	abs	abs
F	Songwe River	-8.9526	33.22449	17-Sep-18	0.5	153	198	29.5	-	0	0	0	abs	abs
G	Nzovwe River	-8.89937	33.3272	17-Sep-18	0.5	374	558	24.3	-	0	0	0	abs	abs
H	Igowilo, Mbeya	-8.89683	33.55588	18-Sep-18	0.1	184	242	24.6	-	0	16	0	abs	2 / 16

–indicates no data, abs = host snails absent

At each of the eight sites, three replicate eDNA samples were collected. Each collection involved the pumping of water though a sterile 0.22 μm Sterivex filter using a sterile 50 ml single-use syringe. Volumes of water filtered varied between 100 to 400 ml (Table 6). After filtration, absolute ethanol was pushed through the filter with a sterile 50 ml syringe to preserve the samples, and filters were kept in labelled 118 ml capacity whirlpak bags. Bottled drinking water was filtered alongside the samples in the field and used as a negative sampling control. DNA was isolated from the filters using the DNeasy Power Water Kit as described above. Each qPCR was performed in triplicate on each eDNA extract, alongside the negative sampling control, the negative non-template PCR control and each of the seven ten-fold serial dilutions of control positive DNA. Analyses of samples for *S*. *mansoni* and *S*. *haematobium* were conducted separately.

**Table 6:** Results of qPCR detection assays of both *S*. *mansoni* and *S*. *haematobium* eDNA from water samples collected in Tanzania 2018.

					*S*. *mansoni*			*S*. *haematobium*		
Site	Name	Replicate	Water sampled (ml)	Total DNA concentration (ng/ul)	Amplification success*	Ct (mean)**	Copies mean (range)***	Amplification success*	Ct (mean)**	Copies mean (range)***
A	Mpemba River	1	400	5.35	1/3	38.5	0.9 (0–2.7)	0/3	-	-
		2	400	2.05	0/3	-	-	0/3	-	-
		3	400	6.85	0/3	-	-	0/3	-	-
B	Mpemba River	1	120	7.60	2/3	39.3	1.02 (0–1.8)	3/3	34.3	9.2(5.6–11.7)
		2	120	7.90	3/3	36.1	19.8 (3.6–29.6)	3/3	33.0	22.7(15.5–31.8)
		3	120	9.35	3/3	39.8	1.7(0.2–2.8)	3/3	34.5	7.7(5.5–11.9)
C	Vwawa River	1	190	2.45	0/3	-	-	0/3	-	-
		2	190	2.10	3/3	43.01	0.12 (0.1–0.2)	0/3	-	-
		3	190	1.30	0/3	-	-	0/3	-	-
D	Mlowo River	1	150	2.80	0/3	-	-	0/3	-	-
		2	150	1.50	1/3	37.48	0.7 (0–5.8)	0/3	-	-
		3	150	3.25	0/3	-	-	0/3	-	-
E	Myovizi River	1	100	1.70	0/3	-	-	0/3	-	-
		2	100	1.25	0/3	-	-	0/3	-	-
		3	100	0.10	0/3	-	-	0/3	-	-
F	Songwe River	1	150	0.70	0/3	-	-	0/3	-	-
		2	150	1.85	0/3	-	-	0/3	-	-
		3	150	1.05	0/3	-	-	0/3	-	-
G	Nzovwe River	1	150	2.45	0/3	-	-	0/3	-	-
		2	150	2.90	0/3	-	-	0/3	-	-
		3	150	0.05	0/3	-	-	0/3	-	-
H	Igowilo, Mbeya	1	160	2.0	0/3	-	-	0/3	-	-
		2	160	3.55	0/3	-	-	0/3	-	-
		3	160	3.80	0/3	-	-	0/3	-	-

*Includes 3 PCRs for each of the 3 samples collected at each site

**includes only the PCR reactions that amplified

***includes all PCR reactions

- Indicates no PCR amplification was present

To quantify the infection status of individual molluscs, a small sample of tissue (no more 20mg) was dissected, and DNA extracted using the DNeasy Blood & Tissue Kit (Qiagen, UK) according to the manufacturer’s protocol. Analyses were conducted using the qPCR approach as described above, although only presence or absence of an amplification is reported herein. We chose a PCR-based screening approach as it allowed us the opportunity to collect the samples in the field and screen the samples at a later date accurately for the focal species; however, the method can amplify individuals with prepatent infections that are not currently shedding cercariae [41]. To confirm the identity of PCR products amplified during these tissue assays, the PCR products from three *Biomphalaria pfeifferi* samples and three *Bulinus globosus* samples were Sanger sequenced. PCR products were purified using DNA Clean & Concentrator-5 (Zymo Research, Irvine, CA, USA) according to the manufacturer’s protocol, and sequenced by Eurofins Genomics (Ebersberg, Germany) using the forward PCR primer. The identity of derived sequences was confirmed using a default BLAST search against the NCBI GenBank nucleotide database.

## Results

### Environmental DNA assays exhibit high levels of species specificity

In qPCR experiments testing the specificity of assays against template genomic DNA, no amplifications from negative controls were observed, and we observed no cross-species amplifications between *S*. *mansoni*, *S*. *haematobium*, *S*. *japonicum*.

When synthetic DNA that included species within the same species group (Fig 1) as the target species was employed, the results indicated some cross-species amplification with the *S*. *haematobium* and *S*. *japonicum* assays albeit with lower levels of qPCR amplification intensity for the non-target species (Table 2). Specifically, the assay for *S*. *japonicum* not only amplified synthetic *S*. *japonicum* DNA (average Ct score 20.19), but also amplified synthetic DNA of closely-related *S*. *mekongi* (average Ct score 28.47). Likewise, the assay for *S*. *haematobium* amplified synthetic *S*. *haematobium* DNA (average Ct score 27.93), but also amplified synthetic DNA of the closely-related *S*. *bovis* (average Ct score 35.67) and *S*. *curassoni* (average Ct score 30.59) (Table 2).

### High sensitivity of *S*. *mansoni* and *S*. *haematobium* eDNA assays

**Fig 3:**
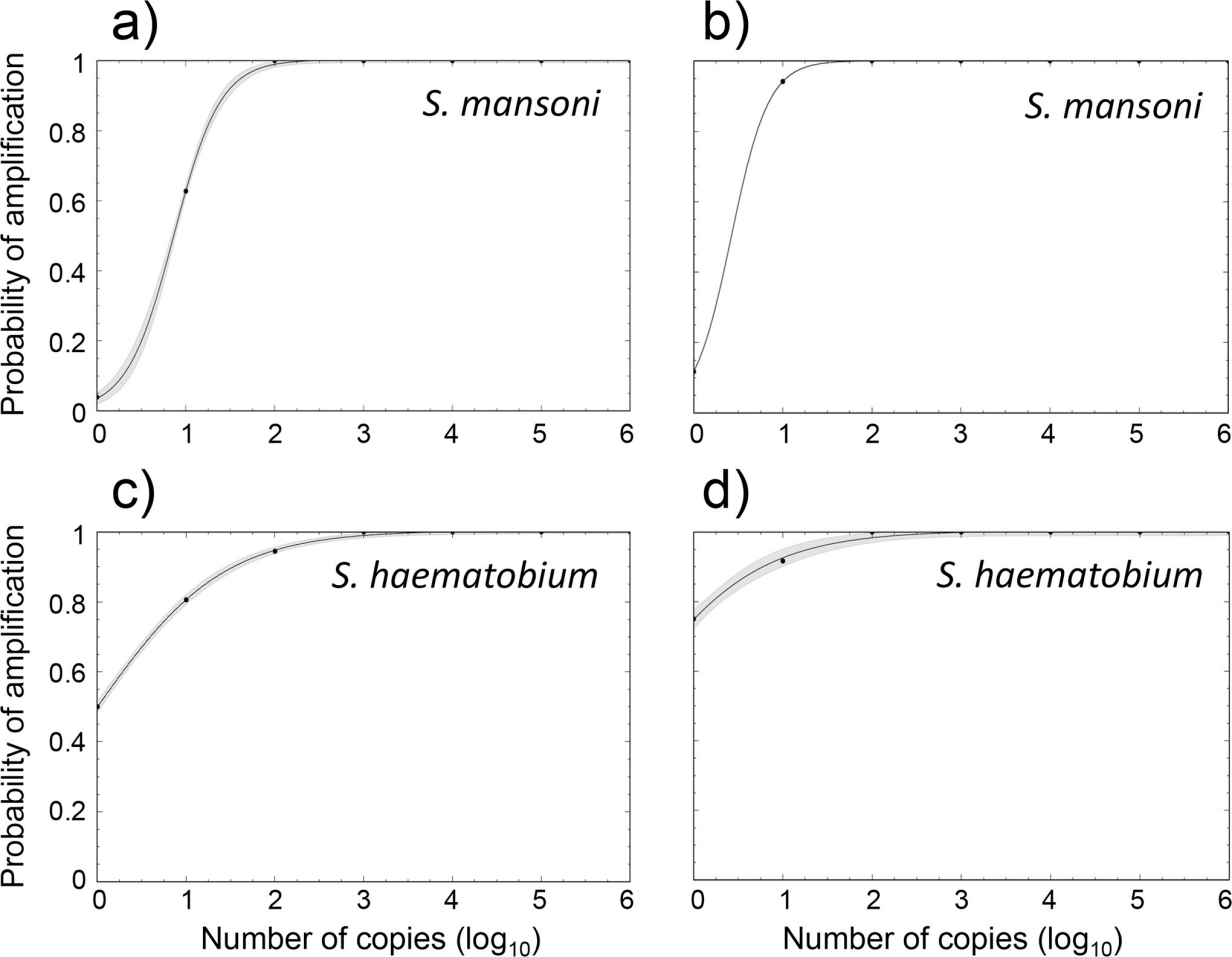
Probability of qPCR amplification of DNA standards of concentrations ranging from 1 copy/μl to 1,000,000 copies/μl. a) *S*. *mansoni* assay–probability of amplification in any one qPCR across all standard templates tested. b) *S*. *mansoni* assay–probability of amplification in any one standard template that is subject to triplicate qPCR, c) *S*. *haematobium* assay–probability of amplification in any one qPCR across all standard templates tested, d) *S*. *haematobium* assay–probability of amplification in any one standard template that is subject to triplicate qPCR. Lines represent logistic models of the form *y* = *a* / (1 + b*e^(-*cx*)^). a) *a* = 1.002, *b* = 27.303, *c* = 3.826. b) *a* = 1.000, *b* = 7.501, *c* = 4.786. c) *a* = 1.004, *b* = 1.009, *c* = 1.415. b) *a* = 1.004, *b* = 0.342, *c* = 1.399. All models *r* > 0.995. Grey shading indicates 95% confidence intervals.

Efficiencies of the *S*. *mansoni* qPCR assay across 17 dilution series ranged from 91.55 to 110.86%, while *R*^2^ ranged from 0.97 to 0.99. The *S*. *mansoni* assay amplified all standards between 1,000,000 copies/μl and 100 copies/μl. Amplification success became inconsistent at 10 copies/μl (32/51 PCR reactions) and was rare at the lowest concentration of 1 copy/ μl (2/51 amplifications). The resolved LOQ for the *S*. *mansoni* assay was therefore 100 copies/μl. The resolved limit of detection where there was 95% probability of successful individual PCR amplification (LOD_I_) was 41.68 copies/μl, while the 95% probability of successful amplification in at least one of the triplicate qPCRs (LOD_III_) was 10.84 copies/μl (Table 3; Fig 3).

Efficiencies of the *S*. *haematobium* qPCR assay across 12 dilution series ranged from 94.87 to 105.45%, while *R*^2^ ranged from 0.98 to 0.99. The *S*. *haematobium* assay amplified all standards between 1,000,000 copies/μl and 1,000 copies/μl. Amplification was mostly successful at 100 copies/μl (34/36 PCR reactions) and 10 copies/μl (29/36 PCR reactions), but was inconsistent at 1 copy/μl (Table 3). The resolved LOQ for the *S*. *haematobium* assay was therefore 100 copies/μl. The resolved limit of detection where there was 95% probability of successful individual PCR amplification (LOD_I_) was 108.39 copies/μl, while the 95% probability of successful amplification in at least one of the triplicate qPCRs (LOD_III_) was 19.19 copies/μl (Table 3; Fig 3).

### Environmental DNA assays detected schistosomes from aquarium water samples

The qPCR assay detected *S*. *mansoni* DNA in all infected aquaria with mean Ct values ranging from 24.64 to 27.77 (Table 4). There were no positive amplifications from non-infected aquaria, or sterilised water controls. Average qPCR efficiency was 103% across the two dilution series assays (range 99.38–106.95) with mean *R*^2^ value of 0.99. All positively amplified samples were above LOQ, LOD_I_ and LOD_III_ values for the species assay, and the mean number of copies of DNA detected in each sample ranged from 7,565 to 48,264 copies/μl (Table 4)

### Environmental DNA detected the presence of schistosomes from the natural environment

Four out of the eight screened sites were positive for *S*. *mansoni* eDNA (sites we named A, B, C and D; Tables 5 & 6, Fig 2). At two of these eDNA positive locations, infected *B*. *globosus* gastropods were present (sites A and B), while at two of the locations *B*. *globosus* was not found (sites C and D; Table 5). Of the four locations that were eDNA negative, only one had *B*. *globosus* present, and these snails were determined to be infected through PCR analysis of tissue DNA (Table 5, Fig 2). Thus, the eDNA assay was congruent with the PCR tests for infected snails in 5/8 (62.5%) of locations. Notably, the estimated numbers of *S*. *mansoni* copies resolved in most samples were below the defined LOQ of 100 copies/μl, the LOD_I_ of 41.68 copies/μl and the LOD_III_ of 10.84 copies/μl.

For *S*. *haematobium*, the target species was detected in eDNA at a single site (Site B, Table 6). At this site all replicates successfully amplified, and infected *Bulinus* snails were abundant (Table 5). Infected *Bulinus* were only detected at one other location (Site A), and this site was resolved as negative for eDNA. Thus, the eDNA assay was congruent with the PCR tests for infected snails in 7/8 (87.5%) of locations. The estimated numbers of *S*. *haematobium* copies were below the defined LOQ of 100 copies/μl, below the LOD_I_ of 108.39 copies/μl, and mainly below the LOD_III_ of 19.19 copies/μl.

Sequencing confirmed the source of PCR product from *B*. *globosus* tissue as *S*. *haematobium*, (100% BLAST match to GenBank accession EU567132). Similarly, the source of PCR product from *B*. *pfeifferi* tissue was confirmed as *S*. *mansoni* (100% BLAST match to GenBank accessions AF130787 and LR214937).

## Discussion

In this study PCR-based assays have been developed to detect the three main human-infecting *Schistosoma* species from tropical freshwaters; *S*. *mansoni*, *S*. *haematobium* and *S*. *japonicum*. Probes and primer pairs were designed to amplify the 16S rRNA region of the mitochondrial DNA, with product size <150 bp which makes them well suited to amplify small fragments of degraded DNA. Our study provided newly characterised primers that reliably amplify and distinguish each of the three main species that infect humans, unlike previous investigations of *Schistosoma* eDNA [32, 33]. In addition, we have exhaustively tested the sensitivity and species-specificity of the assays *in-vitro*. The latter is critical for the evaluation of *Schistosoma* eDNA assays, not only because multiple species within the Schistosomatidae family may be present within the same area, potentially leading to false positive results, but also because *in-silico* testing of primers has frequently been shown to yield inaccurate results when compared to those from *in-vitro* tests [42].

In addition to testing genomic DNA from the focal species, we chose to use synthetic DNA in the *in-vitro* species specificity tests. This approach was selected in order to ensure confidence in the species that the DNA represented, without risk of using natural samples potentially contaminated during their collection and/or curation. Additionally, we were able to test our assays against DNA from a broad variety of relevant species, natural samples of which would be difficult to obtain. Our assays consistently amplified both genomic and synthetic DNA of the target species. However, they also amplified closely-related species. Specifically, the *S*. *haematobium* assay amplified *S*. *bovis* and *S*. *curassoni* from the ‘haematobium group’, while the *S*. *japonicum* assay amplified *S*. *mekongi* from the ‘japonicum group’ (Fig 1). Such cross-species amplification could lead to false-positive amplifications, and in practice the impact of any cross-species amplification will depend on the geographic context where the assay is used. For example, application of the *S*. *haematobium* assay at locations in north-west Africa where it occurs with *S*. *curassoni* and *S*. *bovis* may be affected by false positives. Therefore, during deployment of the assays it may be beneficial to undertake screening to establish *Schistosoma* species present using alternative morphological or genetic methods. Another consideration is that there is potential for cross-species amplification where introgressive hybridization is present among closely-related species, for example between *S*. *haematobium* and *S*. *bovis*, between S. *haematobium* and *S*. *curassoni*, and between *S*. *haematobium* and *S*. *mattheei* [43–45]. In such cases, hybridization can lead to sharing of mitochondrial DNA between parental species, and an environmental DNA assay alone would not be able to distinguish between the species, or identify the presence of hybrids.

We showed it was possible to reliably amplify schistosome eDNA from filters collecting material from natural water bodies and experimental aquaria. None of our negative control samples amplified, and thus we have confidence that the PCRs were amplifying only target schistosome material. It is possible that the filters contained environmental DNA in solution, in cellular debris, and also as whole individual cercariae. From a practical perspective for testing for the presence or absence of the schistosome parasites, it may be of little consequence whether the sample is obtained from partial or whole organisms. However, if the method is to be used for evaluating the relative biomass of schistosomes in the environment, then sampling of whole organisms may be problematic. It is possible that the use of pre-filtration steps may be required to exclude whole organisms, potentially providing more standardised quantitative spatial and temporal comparisons [33]. Alternatively, it would be possible to modify the environmental DNA methods used here to focus sampling exclusively cercariae [46,47]. Such “qPCR cercariometry” requires relatively large volumes of water (e.g. 25 litres) that are filtered through a 20μm mesh zooplankton net, before residual material is collected and concentrated allowing qPCR analyses [46,47]. This cercariometry method would allow the collection of large volumes of genetic material from the environment; however such zooplankton nets are expensive and their re-use requires decontamination between sampling events if they are to remain effective.

Our eDNA assays showed agreement with tests of direct qPCR-based tests of the infection status of snail hosts. Specifically, eDNA showed 62.5% consistency with tests for *S*. *mansoni* in host gastropods, and 87.5% consistency with tests for *S*. *haematobium* in host gastropods. Thus, our assays are similar in performance to those reported by Sengupta *et al*. [33] for *S*. *mansoni* in Kenya with a 71% agreement between eDNA and conventional survey methods. Importantly, both our study and Sengupta *et al*. [33] detected cases where the conventional survey failed to detect evidence of *S*. *mansoni* that was present in the eDNA assay. Such results could have been because schistosomes were present locally, but host snails were rare and therefore difficult to sample. Alternatively, given that the sites at which we detected eDNA but no host snails had flowing water, our analyses could have detected allochthonous schistosome DNA from upstream locations. In either case, the presence of the schistosome eDNA would be indicative of a risk of infection, and perhaps eDNA surveillance could help to identify locations where risk is present but may otherwise go unnoticed. It is also possible that we sampled material from non-transmissive life stages (eggs, miracidia) derived from local contamination sources. Notably, there were also instances where eDNA assays tested negative, but host infected snails were present. In such circumstances it is possible that cercarial production is limited at those locations, or more likely that either eDNA concentrations were below detection limits, or PCR inhibitors were present. Low eDNA concentration is the most likely explanation, however, as the PowerWater extraction kit used has dedicated inhibitor removal steps, and is designed for these kinds of heterogeneous samples. Further simultaneous sampling of both eDNA and host snails is recommended to more reliably estimate the sampling effort required to have high confidence in negative results from these assays.

Multiple studies have described molecular-based diagnostic techniques for *Schistosoma* species [48–51]. These methods, alongside technical improvements in the reliability of PCR, have resulted in an increasing number of research centres in low income countries having access to the real-time PCR diagnostic technology [52]. Widespread uptake of eDNA methods for *Schistosoma* surveillance is therefore feasible in principle. However, the extent to which eDNA methods can replace existing survey protocols will depend on the reliability of the assays for detecting low concentrations of eDNA, as this study identified sites that were eDNA negative but positive by alternative sampling methods. This is most likely because eDNA levels were below detection limits. Specifically, our tests demonstrated that extracted concentrations of schistosome eDNA were at most ~40 copies/ul (which corresponds with ~16 copies/ml of sampled water), which is below our measured limits of detection where individual PCRs becomes 95% reliable. Expectedly, the *S*. *mansoni* assay was most successful at locations A and B where the *Biomphalaria* colonies were large and infection prevalence was extremely high (40% and 88% of snails, respectively). Moreover, the *S*. *haematobium* assay was most successful at the location B where the *Bulinus* colony was large and infection prevalence was as high as 94%. At location A, where the *Bulinus* colony was small yet infection prevalence was 60%, the assay was negative. Thus, the success of the assay is likely to depend both on snail abundance and infection prevalence. Importantly, all the locations where we identified schistosome-positive snails had much higher rates of infection than is typically observed, which can be around 2% [19], although we recognize that the PCR-based tests of snail infection may have revealed cases of prepatent infections in non-shedding snails [41].

Further work is needed to develop improved methods of collecting, preserving and extracting aqueous environmental DNA in tropical environments for schistosome surveillance, including the number and volume of field replicates, the DNA preservation reagents used, and the extraction protocols. Recently Sengupta *et al*. [33] estimated that up to seven one litre water samples may be enough to provide 95% confidence in the presence or absence of *S*. *mansoni*. In this study we used smaller volumes, with the upper limits determined by filter clogging. It may be that more filters are needed to collect the higher volumes of material required for reliable eDNA assays in turbid environments, which are often characteristic of schistosome transmission sites. The characteristics of the environment may also influence the persistence of eDNA and chances of detection [32,53]. For example, suspended sediment can adsorb DNA [54], and the presence of PCR inhibitors such as humic acids, algae and siliceous sediments can determine the success of qPCR-based eDNA assays [55,56]. Therefore further sampling and tests will be required to optimize the eDNA detection power in light of environmental variation. In particular, tests for the presence of PCR inhibition within sampled sites would also be valuable.

To conclude, rapid and inexpensive diagnosis and surveillance of schistosome prevalence in freshwaters will provide timely advice to stakeholders who could promote interventions to interrupt transmission of schistosomiasis in developing countries. As social and environmental change proceeds ever more rapidly, strategies for control of schistosomiasis must increasingly be adapted to local context, and tools for rapid evaluation of parasite presence at fine spatial scales are therefore urgently needed [57]. Our results, together with those of recent parallel studies [32, 33] provide promising indications that eDNA approaches could be considered as a tool for standard monitoring of parasite presence. However, further research is needed to resolve a sampling design that is reliable across habitat types and robust to idiosyncrasies of tropical fieldwork. Moreover, further work is needed to address the shortcomings of the methods we used in terms of specificity and sensitivity. If such limitations could be overcome, the tool could be invaluable for surveillance studies and provide direct support to control strategies for this neglected tropical disease. However, it has been highlighted that the utility of eDNA assays is dependent on the nature of the schistosome life stages present in the environment [58]. More specifically, eDNA assays that yield positive results are unable to distinguish between a site where transmission is active (production of cercariae) or simply contaminated by eggs and miracidia from urine and faeces. Hence, eDNA assays alone may be unable to reliably distinguish locations where transmission has been successfully interrupted by intervention, and in such cases it will need to be coupled with DNA screening of collected snails [58].

## Supporting information

S1 Table Sequences used to develop synthetic DNA for testing species specificity of probes. (DOCX)
