# Correction: Schistosoma species detection by environmental DNA assays in African freshwaters

**DOI:** 10.1371/journal.pntd.0008721

**Published:** 2020-09-01

**Authors:** Hind Alzaylaee, Rupert A. Collins, Gabriel Rinaldi, Asilatu Shechonge, Benjamin Ngatunga, Eric R. Morgan, Martin J. Genner

After publication of this article [1], the authors noticed a reporting error in the results section. The species described in the first paragraph of the results sub-section ‘Environmental DNA detected the presence of schistosomes from the natural environment’ has been reported as *B*. *globosus*, but this should read *B*. *pfeifferi* instead.

The first paragraph of the ‘Environmental DNA detected the presence of schistosomes from the natural environment’ sub-section has now been updated to:

“Four out of the eight screened sites were positive for *S*. *mansoni* eDNA (sites we named A, B, C and D; Tables 5 & 6, Fig 2). At two of these eDNA positive locations, infected *B*. *pfeifferi* gastropods were present (sites A and B), while at two of the locations *B*. *pfeifferi* was not found (sites C and D; Table 5). Of the four locations that were eDNA negative, only one had *B*. *pfeifferi* present, and these snails were determined to be infected through PCR analysis of tissue DNA (Table 5, Fig 2). Thus, the eDNA assay was congruent with the PCR tests for infected snails in 5/8 (62.5%) of locations. Notably, the estimated numbers of *S*. *mansoni* copies resolved in most samples were below the defined LOQ of 100 copies/μl, the LODI of 41.68 copies/μl and the LODIII of 10.84 copies/μl.”

## References

[1] Alzaylaee H, Collins RA, Rinaldi G, Shechonge A, Ngatunga B, Morgan ER, et al (2020) Schistosoma species detection by environmental DNA assays in African freshwaters. PLoS Negl Trop Dis 14(3): e0008129 10.1371/journal.pntd.0008129 32203507 PMC7117781

