# Supplementary material for: Schistosoma species detection by environmental DNA assays in African freshwaters
Source: PLoS Negl Trop Dis. 2020 Mar 23;14(3):e0008129. doi: 10.1371/journal.pntd.0008129 (PMC7117781; doi:10.1371/journal.pntd.0008129)
Supplement: S1 Table — (DOCX) [file pntd.0008129.s001.docx]

**Supporting Information**

S1 Table: Sequences used to develop synthetic DNA for testing species specificity of probes

| Species | Accession number | Sequences | Fragment length | Synthesised DNA quantity | Source |
| --- | --- | --- | --- | --- | --- |
| *S. mansoni* | HE601612 | TTAGTTAATTGTTTAATAGTAAGGCCTGCTCAGTGAAGAAGTTTGTTTAAATAGCCGCGATTATTTATCGTGCTAAGGTAGCATAATATATAGTCTTTTAATTGTAGACTTGTGAATGGTTCAATGAGGTGTGATTAAGGTGATAGTCTATTATCTGAATTTAGTTTAGTGGTTAGGAACCCATTGTTACATTATTAGACGGAAAGACCCCAAGAGCTTTTTT | 223 bp | 2.4 µg | Eurofins Genomics (pEX-A128) |
| *S. rodhaini* | LL973454 | TTAGTTAGTTAGTTAATAGTAAAACCTGCTCAGTGAAATATATATTTTAAATAGCCGCGATTATTGATCGTGCTAAGGTAGCATAATATATAGTCTTTTAATTGTGGACTTGTGAATGGTTCAACGAGGTGTGATTAAGGTGATAGTTTGTTTCTGAATTTAGTTTGGTGGTTAAGAACCCACTATTATAATATTAGACGGAAAGACCCCAAGAGCTTTTCT | 222 bp | 5.1 µg | Eurofins Genomics (pEX-A128) |
| *S. spindale* | DQ157223 | TTTGTGTAAGAGGTAAATAGTATGACCTGCCCAATGAACAGATATATGAATGGCCGCAGCTTTTCGCTGTGCTAAGGTAGCATAATATATAGTTTTTTAATTGGGGACTTGTGAATGGTTTAATGAAGGGTGTCTAAAATAATAATTATTTCTGAAATTAATTTAGTGGTAAGGAATCCACTATTAAGATATAGGACGGAAAGACCCCAAGAGCTTTACA | 220 bp | 5 µg | Invitrogen by Thermo-Fisher Scientific (pMA-T) |
| *S. incognitum* | EF534285 | TTTGTTAGATATAAATGATATGACCTGCTCAATGTAGAAGTGTATAAATGGCCGCGGTTAAGAGTTTCGTGCTAAGGTAGCATAATATATAGCTTTTTAATTGAGGGCTTGTGAATGGTTTAATGAGATTTACATAAAAGGGCGATCTTTTTTGAAATTGATTTATTAGTTAAGAATCTGATATTATAATATAAGACGGAAAGACCCCGAGATCTTTACT | 220 bp | 5 µg | Invitrogen by Thermo-Fisher Scientific (pMA-RQ (AmpR) |
| *S. haematobium* | DQ157222 | TCTGATGAGGTTTAGATAGTATGACCTGCTCGATGAAAATGAACATGAATGGCCGCAGCTTTAGCTGTGCTAAGGTAGCATAATATATAGTTTTTTGATTGGAGACTTGTGAATGGTCGAACGAAAGGTGTCTAAAATGATAATTATGTCTGAATTTAGTTTAAGTGGTGAGGAACCCATTTTTTGATTATAGGACGGAAAGACCCCAAGAGTTTTACT | 219 bp | 5 µg | Invitrogen by Thermo-Fisher Scientific (pMA-T) |
| *S. bovis* | QMKO01004774 | TCTGATGAGGTTTAGATAGTATGACCTGCCCAATGAAAGTAAACATGAATGGCCGCAGCTTTAGCTGTGCTAAGGTAGCATAATATATAGTTTTTTGATTGGAGACTTGTGAATGGTTGAACGAAAGGTGTCTAAAATGATAATTATATCTGAATTTAGTTTAGTGGTGAGGAATCCATTATTTAAATATAGGACGGAAAGACCCCAAGAGTTTTACT | 218 bp | 2.9 µg | Eurofins Genomics (pEX-A128) |
| *S. curassoni* | AP017708 | TCTGATGAGATTTAGATAGTATGACCTGCCCAATGAAAGTAAACATGAATGGCCGCAGCTTTAGCTGTGCTAAGGTAGCATAATATATAGTTTTTTGATTGGAGACTTGTGAATGGTTGAACGAAAGGTGTCTAAAATGACAATTATATCTGAATTTAGTTTAGTGGTGAGGAACCCATTATTTAGATATAGGACGGAAAGACCCCAAGAGTTTTACT | 218 bp | 5 µg | Invitrogen by Thermo-Fisher Scientific (pMA-T) |
| *S. margrebowiei* | AP017709 | TTTTGGTAGATTTAAATAGTATGACCTGCCCAATGAAACTTAACATGAATGGCCGCGGCATTAGCCGTGCTAAGGTAGCATAATATATAGTTTTTTAATTGGAGACTTGTGAATGGTTGAACGAAAGGTGTCTAAAATGGTAACTGTATCTGAATTTAGTTGAGCGGTGAGGAACCCGTTGTTATAGTATAGGACGGAAAGACCCCAAGAGTTTTACT | 218 bp | 5 µg | Invitrogen by Thermo-Fisher Scientific (pMA-T) |
| *S. indicum* | EF534284 | TTTGGGAGAGATAAATAGTATGACCTGCCCAATGAGCTTTTAAATGAATGGCCGCGGCTTTAGTCGTGCTAAGGTAGCATAATATATAGTTTTTTAATTGGAGACTTGTGAATGGTTTAATGAAAGGTGTCTAAAATGATAGCTGTTTCTGAATTTAGTACGGTGGTCAGGAATCCATTGTTGAGATATAGGACGGAAAGACCCCAAGAGCTTTACA | 217 bp | 5 µg | Invitrogen by Thermo-Fisher Scientific (pMA-T) |
| *S. japonicum* | JQ781206 | TTTGTTGGATTTAAATAGTATGGCCTGCCCAATGTTGTAAATTAATGGTCGCAGTTTTACTGTGCTAAGGTAGCATAATATATCGCTTCTTAATTAGTGGCTTGTGAATGGTTTAATGAAATAGAATATTTAAATGATGACTATATCTGAAATTGATTTAGTGGTGCGGAATCCATTGTTATAGTATAAGACGGAAAGACCCCGAGATCTTGAATT | 216 bp | 1.5 µg | Invitrogen by Thermo-Fisher Scientific (pMA-T) |
| *S. mekongi* | AF217449 | TTTGTTAGGTTTAAATAGTATGGCCTGCCCACTGTTGGAATAAATGGTCGCAGATTTTCTGTGCTAAGGTAGCATAATATATCGCTTCTTAATTAGTGGCTTGTGAATGGTTTAATGAAATAGGGTGCTTAAATGATGACTATTTCTGAAATTGGTTTAGTGATGAGGAACTCGCTGTGATAATATAAGACGGAAAGACCCCAAGATCTTGGATT | 215 bp | 3 µg | Invitrogen by Thermo-Fisher Scientific (pMA-T) |
